# Micro-PET imaging of hepatitis C virus NS3/4A protease activity using a protease-activatable retention probe

**DOI:** 10.3389/fmicb.2022.896588

**Published:** 2022-11-04

**Authors:** Chih-Hung Chuang, Tian-Lu Cheng, Wei-Chun Chen, Yi-Jung Huang, Hsin-Ell Wang, Yen-Chen Lo, Yuan-Chin Hsieh, Wen-Wei Lin, Ya-Ju Hsieh, Chien-Chih Ke, Kang-Chieh Huang, Jin-Ching Lee, Ming-Yii Huang

**Affiliations:** ^1^Department of Medical Laboratory Science and Biotechnology, Kaohsiung Medical University, Kaohsiung, Taiwan; ^2^Drug Development and Value Creation Research Center, Kaohsiung Medical University, Kaohsiung, Taiwan; ^3^College of Medicine, Graduate Institute of Medicine, Kaohsiung Medical University, Kaohsiung, Taiwan; ^4^Department of Biomedical and Environmental Biology, Kaohsiung Medical University, Kaohsiung, Taiwan; ^5^Department of Marine Biotechnology and Resources, National Sun Yat-sen University, Kaohsiung, Taiwan; ^6^Department of Biomedical Imaging and Radiological Sciences, National Yang-Ming University, Taipei City, Taiwan; ^7^School of Medicine for International Students, I-Shou University, Kaohsiung, Taiwan; ^8^Department of Laboratory Medicine, School of Medicine, Kaohsiung Medical University, Kaohsiung, Taiwan; ^9^Department of Medical Imaging and Radiological Sciences, Kaohsiung Medical University, Kaohsiung, Taiwan; ^10^Department of Biotechnology, College of Life Science, Kaohsiung Medical University, Kaohsiung, Taiwan; ^11^Department of Radiation Oncology, Kaohsiung Medical University Hospital, Kaohsiung Medical University, Kaohsiung, Taiwan; ^12^Department of Radiation Oncology, School of Medicine, College of Medicine, Kaohsiung Medical University, Kaohsiung, Taiwan; ^13^Center for Cancer Research, Kaohsiung Medical University, Kaohsiung, Taiwan

**Keywords:** cellular protease activity, HCV NS3/4A serine protease, protease-activated retention peptide, micro-positron emission tomography, TAT-ΔNS3/4A-^124^I-FITC probe

## Abstract

Hepatitis C virus (HCV) NS3/4A protease is an attractive target for direct-acting antiviral agents. Real-time tracking of the NS3/4A protease distribution and activity is useful for clinical diagnosis and disease management. However, no approach has been developed that can systemically detect NS3/4A protease activity or distribution. We designed a protease-activatable retention probe for tracking HCV NS3/4A protease activity *via* positron emission topography (PET) imaging. A cell-penetrating probe was designed that consisted of a cell-penetrating Tat peptide, HCV NS3/4A protease substrate, and a hydrophilic domain. The probe was labeled by fluorescein isothiocyanate (FITC) and ^124^I in the hydrophilic domain to form a TAT-ΔNS3/4A-^124^I-FITC probe. Upon cleavage at NS3/4A substrate, the non-penetrating hydrophilic domain is released and accumulated in the cytoplasm allowing PET or optical imaging. The TAT-ΔNS3/4A-FITC probe selectively accumulated in NS3/4A-expressing HCC36 (NS3/4A-HCC36) cells/tumors and HCV-infected HCC36 cells. PET imaging showed that the TAT-ΔNS3/4A-^124^I-FITC probe selectively accumulated in the NS3/4A-HCC36 xenograft tumors and liver-implanted NS3/4A-HCC36 tumors, but not in the control HCC36 tumors. The TAT-ΔNS3/4A-^124^I-FITC probe can be used to represent NS3/4 protease activity and distribution *via* a clinical PET imaging system allowing. This strategy may be extended to detect any cellular protease activity for optimization the protease-based therapies.

## Introduction

The World Health Organization (WHO) estimates that worldwide, at least 170 million people (3% of the world population) are infected with hepatitis C virus (HCV) (Wasley and Alter, 2000; Kondou, 2022). HCV infection often leads to chronic infection and is associated with high risk of the development of liver cirrhosis and hepatocellular carcinoma (Jahan et al., 2012; Fu et al., 2018; Glitscher et al., 2022; Yoo et al., 2022). For example, HCV NS3/4A protease highly expressed in HCV-infected patients is required for viral replication (Tomei et al., 1993) and virus particle assembly (Phan et al., 2011). The NS3/4A protease activity has been reported that is highly associated with the development of liver cirrhosis and hepatocellular carcinoma (McGivern and Lemon, 2009; Presser et al., 2013; Sakata et al., 2013). Sakata and colleagues indicated that the HCV NS3/4A protease enhances liver fibrosis *via* binding to and activating TGF-β type I receptor in HCV-infected chimeric mice (Laurent et al., 2013). Given their relevance, NS3/4A proteases is attractive targets for the design of antiviral drugs (Cai et al., 2020). Currently, the boceprevir and telaprevir are the protease inhibitors and that are approved by FDA for the treatment of HCV genotype 1 infected patients (Laurent et al., 2013; Deeks, 2014). Furthermore, many clinical studies have reported that blocking the NS3/4A protease activity using a protease inhibitor significantly reduces the virus load in patients (Lamarre et al., 2003; Salam and Akimitsu, 2013; Hayashi et al., 2014), indicating that the HCV NS3/4A protease activity may be a useful marker to predict HCV viral activity and disease progression (Pan et al., 2019; Kim et al., 2021). Therefore, the technology to tracking the NS3 protease activity *in vivo* would provide a powerful tool to design the personalized protease inhibitor-based therapies and to monitor the development of liver cirrhosis and hepatocellular carcinoma (Hasegawa et al., 2015).

**FIGURE 1:**
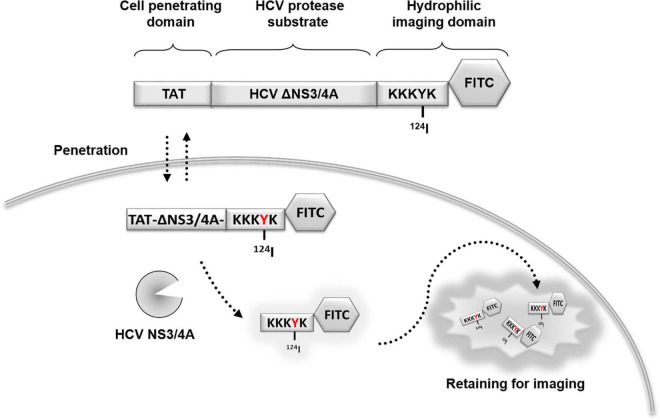
Schematic representation of the protease-activated retention micro-PET probe. Hepatitis C virus (HCV) tracking probe was designed using HCV NS3/4A serine protease as a marker of viral activity. A cell-penetrating probe was designed that consisted of a cell-penetrating Tat peptide, a HCV NS3/4A protease substrate, and a hydrophilic domain. For imaging, the probe was labeled by fluorescein FITC and ^124^I in the hydrophilic domain to form a TAT-ΔNS3/4A-^124^I-FITC probe. Upon cleavage at NS3/4A substrate, the non-penetrating hydrophilic domain is released and accumulated in the cytoplasm allowing visualization using PET or optical imaging. NS, non-structural protein; TAT, transactivator of transcription of human immunodeficiency virus; FITC, fluorescein isothiocyanate; HCV, hepatitis C virus; PET, positron emission tomography.

However, the current approaches to detect HCV NS3/4A protease activity *in vivo* were not sufficient. For example, several cell-based systems for monitoring NS3/4A activity have been reported (Lee et al., 2003; Boonstra et al., 2009). However, these cell-based systems cannot assess the efficacy, toxicity, and bioavailability of HCV NS3/4A protease inhibitors *in vivo*. To overcome this problem, Wang and colleagues developed a stable Huh7-[ANLuc(NS5A/B)BCLuc] cell line that can report the NS3/4A serine protease activity *via* bioluminescence imaging in living animals (Wang et al., 2010). However, the low tissue penetration of the luminescent signal limits these reporters to studies of small animals. Currently, the chimpanzee is still the animal of choice for the study of antiviral therapy against HCV (Abe et al., 1992), meaning that a higher sensitivity and more penetrative imaging probe is necessary. To overcome these challenges, we developed a PET probe that consisted of a cell-penetrating Tat peptide (GRKKRRQRRRPQ) (Vives et al., 1997), HCV NS3/4A protease substrate (DEDEDEDEMEECASHL) (Lee et al., 2003), and the hydrophilic domain (KKKYK). The probe was labeled by FITC and ^124^I in the hydrophilic domain to generate the cell-permeable probe (TAT-ΔNS3/4A-^124^I-FITC). Upon cleavage at NS3/4A substrate, the non-penetrating hydrophilic domain is released and accumulated in the cytoplasm and can be visualized by PET or optical imaging (Figure 1). We first examined whether TAT-ΔNS3/4A-FITC could selectively accumulate at sites with protease activity, including protease NS3/4A-expressing HCC36 (NS3/4A-HCC36) cells, HCV-infected HCC36 cells, and NS3/4A-containing HCC36 tumors. We also examined whether the TAT-ΔNS3/4A-^124^I-FITC probe could be selectively retained in NS3/4A-HCC36 tumors in xenograft mice by micro-PET imaging. The radioactivity in selected tissues was then examined to quantify the probe biodistribution. Finally, we evaluated whether this probe could accumulate in NS3/4A-HCC36 liver-implanted mice through micro-PET imaging of mice. The successful development of a clinically available PET probe to represent NS3/4A activity would provide a valuable tool for real-time tracking of protease activity and distribution.

## Materials and methods

### Cells and animals

Human hepatocellular carcinoma cells HCC36 (American Type Culture Collection, Manassas, VA, United States) were cultured in Dulbecco’s Minimal Essential Medium (Sigma-Aldrich, Burlington, MA, United States) supplemented with 10% heat-inactivated bovine calf serum, 100 units/ml penicillin, and 100 μg/ml streptomycin (Sigma-Aldrich, Burlington, MA, United States) at 37°C in a humidified atmosphere of 5% CO2. NS3/4A-HCC36 cell lines by transfection of recombinant lentivirus consist package of plasmid: pCMVΔR8.91 (RNAi core, Academia Sinica, Taipei, Taiwan) and pMD.G (RNAi core, Academia Sinica, Taipei, Taiwan) and target plasmid: pLKO_AS3 NS3/4A, respectively; after selection by 1, 3, and 5 μg/ml puromycin dihydrochloride (P8833, Sigma-Aldrich, Burlington, MA, United States), the selection medium was replaced every 2 days for 1 week. Female BALB/c nude mice (6–8 weeks old) were obtained from the National Laboratory Animal Center, Taipei, Taiwan. All animal experiments were performed in accordance with institutional guidelines and approved by the Animal Care and Use Committee of Kaohsiung Medical University.

### Synthesis of TAT-ΔNS3/4A-FITC

TAT-ΔNS3/4A-FITC (GRKKRRQRRRDEDEDEDEMEEC ASHLKKKYK-FITC) and scrambled sequence probe (GRKKRRQRRR-ECEEEEESMDDDAH-LKKKYK) were synthesized by Kelowna International Scientific (Taipei, Taiwan) and purified to > 95% purity by high-performance liquid chromatography [Merck Hibar C18 column, 4 × 250 mm; eluted at 1 mL/min with a gradient starting from 95% solvent A (0.1% trifluoroacetic acid in water) and 5% solvent B (0.1% trifluoroacetic acid in MeCN) to 5% solvent A and 95% solvent B at 30 min].

### Specific retention of TAT-ΔNS3/4A-FITC in NS3/4A-expressing cells

HCC36 or NS3/4A-HCC36 cells (1 × 10^5^) were seeded with DMEM containing 10% serum in a 24-well plate at 37°C in a CO_2_ incubator overnight. The cells were incubated with 10 μM of TAT-ΔNS3/4A-FITC in the presence or absence of 2 μM telaprevir (purchased from Legend Star International, Taiwan) at 37°C for 1 h. After probe staining, the cells were washed using DMEM containing 10% serum per 20 min until the end of the experiment. After 8 h washing, the fluorescence of viable cells was observed under a fluorescence microscope at the indicated times (Axiovert 200, Carl Zeiss MicroImaging, GmbH, Germany).

### Specific retention of TAT-ΔNS3/4A-FITC in hepatitis C virus-infected cells

Infectious HCV particles were generated as previously described (Hayashi et al., 2014). HCC36 cells (1 × 10^5^) were seeded in DMEM containing 10% serum in a 24-well plate at 37°C in a CO_2_ incubator overnight. The cells were infected with JFH-1 and incubated with or without 2 μM telaprevir. After 5 days, the parental HCC36 and JFH-1-infected cells were stained with 10 μM of TAT-ΔNS3/4A-FITCat 37*^o^*C for 1 h. After probe staining, the cells were washed using DMEM containing 10% serum per 20 min until the end of the experiment. After 8 h washing, the fluorescence of viable cells was observed under a fluorescence microscope at the indicated times (Axiovert 200, Carl Zeiss MicroImaging, GmbH, Germany).

### Histological analysis of the fluorescence intensity and protease activity in tumors

BALB/c nude mice (*n* = 3) bearing established HCC36 and NS3/4A-HCC36 (100–200 mm^3^) tumors in their right and left hind legs, respectively, were intravenously injected with 500 μM (in 100 μL) TAT-ΔNS3/4A-FITC and sacrificed 4 h later. Tumors were excised and embedded in OCT compound (Tissue-Tek, CA) in liquid nitrogen. The adjacent tumor sections either directly detect the FITC-fluorescent intensity to observe the accumulation of TAT-ΔNS3/4A-FITC probe or stained with a 520 HCV protease Assay Kit (AnaSpec) to visualize NS3/4A activity. The sections were examined on an upright BX4 microscope (Olympus, Melville, NY, USA) or viewed under phase contrast or fluorescence fields on an inverted Axiovert 200 microscope (Carl Zeiss Microimaging, Thornwood, NY, USA).

### Synthesis and purification of TAT-ΔNS3/4A-^124^I-FITC

^124^I was purchased from IBA Molecular, VA, USA (2.6 TBq/mL, n.c.a.). Radio-iodination (nucleophilic aromatic addition) was performed by adding Na^124^I (2.6 TBq/mL, no carrier added) into TAT-ΔNS3/4A-FITC (0.5 mg, MW = 4339.8) with hydrogen peroxide solution (H_2_O_2_: HCl: H_2_O = 16:16:68) as an oxidant under condition. The solution was reacted at room temperature for 10 min with vigorous vortexing, followed by adding 2 mM sodium thiosulfate solution and saturated sodium hydrogen carbonate to neutralize the solution. TAT-ΔNS3/4A-^124^I-FITC was purified by C-18 sep-pak (Waters, Milford). The radiochemical purity of TAT-ΔNS3/4A-^124^I-FITC was determined using HPLC (Delta 600, Waters). For HPLC analysis, a reverse phase column (Purospher STAR RP-18e, 10 × 250 mm, MERCK, Darmstadt, Germany) was used and eluted with acetonitrile (ACN)/0.1% TFA in water (3/97, v/v) at a flow rate of 4 mL/min.

### Specificity and serum half-life of TAT-ΔNS3/4A-^124^I-FITC

HCC36 or NS3/4A-HCC36 (1 × 10^5^) cells were seeded with DMEM containing 10% serum in a 24-well plate at 37°C in a CO_2_ incubator overnight. The cells were incubated with 37 kBq of TAT-ΔNS3/4A-^124^I-FITC in the presence or absence of 2 μM telaprevir (Vertex, Cambridge MA) at 37°C for 1 h. After probe staining, the cells were washed using DMEM containing 10% serum per 20 min until the end of the experiment. At different time points, the cells were collected by treatment with trypsin. The radioactivity of the cells was then measured using a gamma-counter. The CPM was normalized by protein concentration.

BALB/c nude mice (*n* = 3) were intravenously injected with 3,700 kBq TAT-ΔNS3/4A-^124^I-FITC, and blood samples were periodically removed from the tail vein of the mice. The blood was weighed on an analytical balance and assayed for radioactivity in a multichannel gamma-counter. The initial and terminal half-life of the probe was estimated by fitting the data to a two-phase exponential decay model with Prism 4 software (GraphPad Software, San Diego, CA, USA).

### Micro-PET imaging of NS3/4A activity *in vivo*

BALB/c nude mice (*n* = 3) bearing established HCC36 and NS3/4A-HCC36 (100–200 mm^3^) in their right and left hind leg, respectively, were anesthetized by halothane vapor with a vaporizer system. The mice were feed the Lugol’s solution and then intravenously injected with 3,700 kBq (in 100 μL) TAT-ΔNS3/4A-^124^I-FITC. PET imaging was sequentially performed at 2, 4, and 6 h. To test the specificity of TAT-ΔNS3/4A-^124^I-FITC, telaprevir (20 mg/kg/day for 3 days) or control vehicle was intraperitoneally injected into mice (*n* = 3) 3 days prior to TAT-ΔNS3/4A-^124^I-FITC.

The orthotopic liver implantation model was generated as previously described (Kim et al., 2009). The NS3/4A-HCC36 tumors or HCC36 tumors from the ectopic tumors were harvested and transplanted into the left liver lobe of SCID mice (n = 3). After 2 weeks, the mice were feed the Lugol’s solution, and then, the 3,700 kBq of TAT-ΔNS3/4A-^124^I-FITC was intravenously injected.

The mice were positioned in a micro-PET scanner (R4; Concorde Microsystems, Knoxville, Tenn) with their long axis parallel to the transaxial plane of the scanner. The scanner has a computer-controlled bed with a 10.8-cm transaxial and 8-cm axial field of view. It has no septa and operates exclusively in a three-dimensional list mode. All raw data were first sorted into three-dimensional sinograms, followed by Fourier rebinning and ordered-subsets expectation maximization image reconstruction. Fully three-dimensional list-mode data were collected by using an energy window of 350–750 keV and a time window of 6 nsec. Image pixel size was 0.85 mm transaxially, with a 1.21-mm section thickness. The region of interest was analyzed with ASIPro VM version 5.0 (Concorde Microsystems, Knoxville, TN, USA) analysis software.

### Biodistribution of TAT-ΔNS3/4A-^124^I-FITC *in vivo*

In xenograft model, BALB/c nude mice bearing established NS3/4A-HCC36 and HCC36 tumors (*n* = 3) were intravenously injected with 3,700 kBq (in 100 μL) TAT-ΔNS3/4A-^124^I-FITC. In orthotopic model, SCID mice (*n* = 3) with NS3/4A-HCC36 or HCC36 tumors implanted in the liver were intravenously also injected with 3,700 kBq (in 100 μL) TAT-ΔNS3/4A-^124^I-FITC. Animals were killed after anesthesia with pentobarbital (65 mg/kg) at 2, 4, and 6 h. Radioactivity in isolated tumors and tissues was measured with a multichannel gamma-counter. The biodistribution of the probe was expressed as percentage injected dose per gram of tissue (%ID/g).

### Statistical analysis

Statistical significance of differences between mean values was estimated with InStat software (version 3.0; GraphPad software) using the independent Student’s *t*-test for unequal variances. *P*-values of less than 0.05 were considered statistically significant.

## Results

### Generation of NS3/4A-expressing cells

To setup NS3/4A stably expressing cells to visualize NS3/4A protease activity *in vitro* and *in vivo*, we constructed the NS3/4A gene (HCV JFH-1 2a strain) into a lentiviral vector, pLKO_AS3 NS3/4A, and the infected cells were selected *via* puromycin to directly express HCV NS3/4A protease in the human hepatocellular carcinoma HCC36 cells (NS3/4A-HCC36). The expression of NS3/4A protease was confirmed by Western blot using anti-NS3 antibodies (ab13830, Abcam UK). As shown in Supplementary Figure 1A, NS3/4A protease could be detected in the NS3/4A-HCC36 cells and HCV replicon-containing cell line (AVA5) (Lee et al., 2004) but not in the control HCC36 cells. In addition, these cells were transfected with the NS3 response reporter vector pEG-(DEΔ4AB)-SEAP, containing the NS4A/B junction between egfp and seap, to detect the NS3/4A activity (Lee et al., 2003). Upon NS3/4A cleavage, the secreted embryonic alkaline phosphatase (SEAP) is secreted. The activity of NS3/4A protease can be quantitatively indicated by measuring the SEAP activity in the cell culture medium. Strong SEAP activity could be detected in the NS3/4A-HCC36 cells and HCV replicon-containing cell line (AVA5) but not in the control HCC36 cells (Supplementary Figure 1B), indicating strong NS3/4A activity in the NS3/4A-HCC36 cells. These results indicated the NS3/4A protease is stable and functionally expressed in NS3/4A-HCC36 cells.

### Specific retention of TAT-ΔNS3/4A-FITC in NS3/4A-HCC36-and hepatitis C virus-infected cells

**FIGURE 2:**
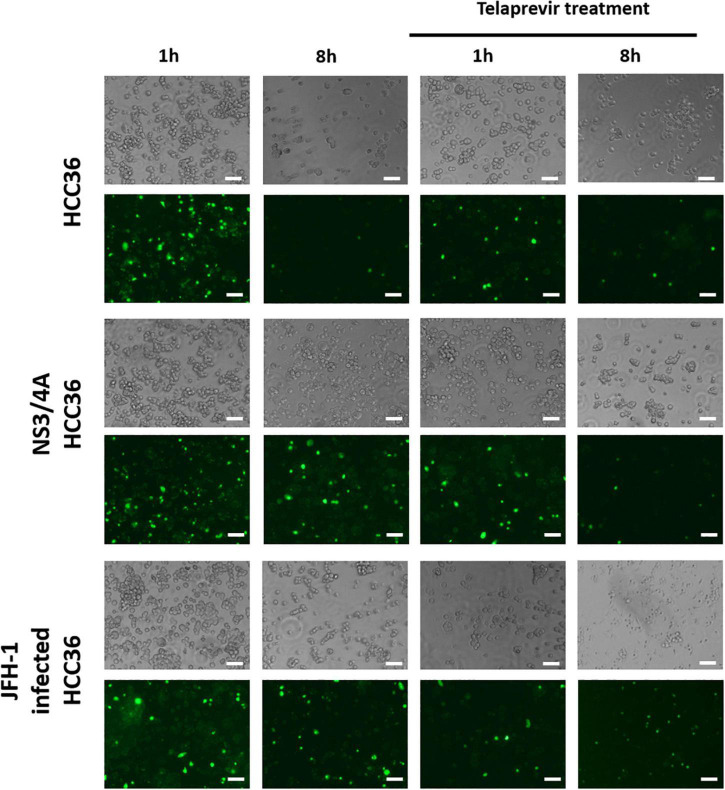
Specific retention of TAT-ΔNS3/4A-FITC in NS3/4A-HCC36- and HCV-infected cells. HCC36 cells, NS3/4A-HCC36 cells, or JFH-1-infected HCC36 cells were incubated with 10 μM TAT-ΔNS3/4A-FITC in the absence or presence of 2 μM telaprevir (NS3/4A protease inhibitor) at 37°C for 1 h. The cells were washed with DMEM containing 10% serum three times per hour. After culturing for 1 or 8 h, the phase contrast and fluorescence of viable cells were observed under a fluorescence microscope. Scale bar: 100 μm.

To evaluate whether this protease-activated retention probe (TAT-ΔNS3/4A-FITC) could specifically accumulate and be retained in the NS3/4A-expressing cells, we incubated the HCC36 cells, NS3/4A-HCC36 cells, or HCV (JFH-1)-infected HCC36 cells with for 1 h. After culturing for 8 h, the accumulation of the fluorescence signal was observed using a fluorescence microscope. Figure 2 shows that strong fluorescence accumulated in each cell after probe staining. After culturing for 8 h, the strong fluorescence only accumulated in NS3/4A-HCC36 cells and HCV-infected HCC36 cells but not in control HCC36 cells. We also compared the NS3/4A protease activity between NS3/4A-HCC36 cells and JFH-1-infected HCC36 cells. The relative protease activity of HCC36 cells, NS3/4A-HCC36 cells, and JFH-1-infected HCC36 cells was 1.27 ± 0.18, 7.09 ± 0.29, and 5.64 ± 0.22, respectively (Supplementary Figure 2). These results indicated that the TAT-ΔNS3/4A-FITC probe selectively accumulated and retained the fluorescence signal in the NS3/4A-expressing cells or virus-infected cells.

To confirm that the accumulation of TAT-ΔNS3/4A-FITC is dependent on protease activity, we incubated the HCC36 cells, NS3/4A-HCC36 cells, or HCV (JFH-1)-infected HCC36 cells with the probe in the presence of NS3/4A inhibitor (Telaprevir, VX-950) (Salam and Akimitsu, 2013). After culturing for 8 h, the accumulation of fluorescence signals was observed under a fluorescence microscope. The fluorescence of the probe did not accumulate in the NS3/4A-HCC36 cells or HCV (JFH-1)-infected HCC36 cells after inhibition, indicating that accumulation of TAT-ΔNS3/4A-FITC probe is dependent on protease activity (Figure 2).

We also used the WT probe (GRKKRRQRRR-DEDEDEDEMEECASH-LKKKYK-FITC) and the scrambled sequence probe (GRKKRRQRRR-ECEEEEESMDDDAH-LKKKYK) that could not be activated by protease as a control group to test the specific retention of probe *via* flow cytometry. HCC36 cells, NS3/4A-HCC36 cells, or JFH-1-infected HCC36 cells were incubated with 10 μM TAT-ΔNS3/4A-FITC or 10 μM TAT-scrambled NS3/4A-FITC in the absence or presence of 2 μM telaprevir (NS3/4A protease inhibitor) at 37oC for 1 h. The cells were washed with DMEM containing 10% serum three times per hour. After culturing for 1 or 8 h, the fluorescence of viable cells was observed *via* flow cytometry. The Supplementary Figure 5 shows that strong fluorescence of WT probe accumulated in each cell after 1 h probe staining. After culturing for 8 h, the strong fluorescence of WT probe only accumulated in NS3/4A-HCC36 cells and HCV-infected HCC36 cells but not in control HCC36 cells. In addition, the strong fluorescence of WT probe (8 h) could block *via* the 2 μM telaprevir (NS3/4A protease inhibitor). The fluorescence of scrambled sequence probe cannot be accumulated in each cell after 8-h probe staining. These results infected the accumulation of TAT-ΔNS3/4A-FITC probe is dependent on protease activity.

### *In vivo* accumulation of TAT-ΔNS3/4A-FITC in NS3/4A-expressing tumors

**FIGURE 3:**
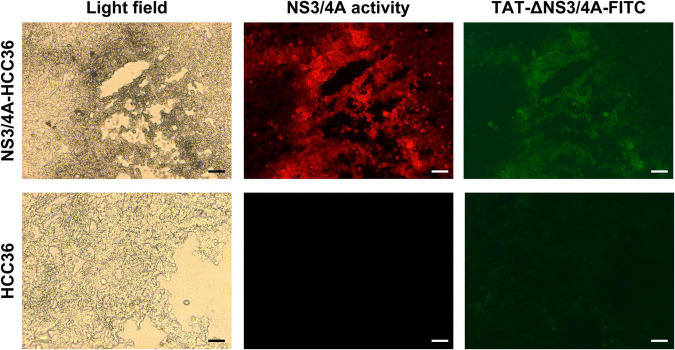
Specific retention of TAT-ΔNS3/4A-FITC in NS3/4A-expressing tumors *in vivo*. Mice bearing established NS3/4A-HCC36 and HCC36 tumors were injected with 500 μM TAT-NS3/4A-FITC and sacrificed after 4 h. Sections of NS3/4A-HCC36 (upper panels) and HCC36 (lower panels) tumors were stained with 520 HCV protease Assay Kit (AnaSpec) to detect NS3/4A activity in tumor sections. FITC-derived fluorescence (green) and NS3/4A activity (red) were observed under a fluorescence microscope. Scale bar: 1 mm.

To examine whether the TAT-ΔNS3/4A-FITC probes can specifically detect NS3/4A activity *in vivo*, NS3/4A-HCC36 and HCC36 tumors were frozen sectioned at 4 h after the injection of TAT-ΔNS3/4A-FITC probes in tumor-bearing mice. The adjacent tumor sections either directly detect the FITC-fluorescent intensity to observe the accumulation of TAT-ΔNS3/4A-FITC probe or stained with NS3/4A substrate to visualize NS3/4A activity. FITC-derived fluorescence was accumulated in the NS3/4A-HCC3 tumors, but not the control HCC36 tumors, which was matched with red fluorescence for NS3/4A activity (Figure 3). These results indicated that the retention of AT-ΔNS3/4A-FITC probe correlated with NS3/4A activity *in vivo*.

### Specificity and half-life of TAT-ΔNS3/4A-^124^I-FITC

We labeled TAT-ΔNS3/4A-FITC with ^124^I to generate a TAT-ΔNS3/4A-^124^I-FITC probe. A representative analytical HPLC chromatogram of the TAT-ΔNS3/4A-^124^I-FITC probe was shown in Supplementary Figure 3. The retention time of ^124^I and TAT-ΔNS3/4A-^124^I-FITC was ∼4.7–5 and 6.7 min, respectively. The crude TAT-ΔNS3/4A-^124^I-FITC was purified by using a C-18 sep-pak (Waters, Milford) separation unit. After purification, the radiochemical purity (RCP) of TAT-ΔNS3/4A-^124^I-FITC was higher than 95% (Supplementary Figure 3C) (radio-peak, 6.7 min). The radiochemistry yield is about 25–55%.

**FIGURE 4:**
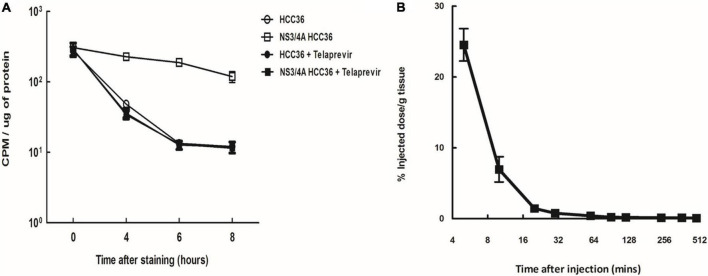
Specificity and half-life of TAT-ΔNS3/4A-^124^I-FITC. **(A)** NS3/4A-HCC36 and HCC36 cells were incubated with 37 kBq of TAT-ΔNS3/4A-^124^I-FITC in the presence or absence of 2 μM Telaprevir at 37°C for 1 h. The cells were washed with DMEM containing 10% serum three times per hour. The cells were collected by treatment with trypsin at the indicated times. The radioactivity of the cells was then measured with a gamma-counter. The CPM was normalized by protein concentration. **(B)** Kinetics of the TAT-ΔNS3/4A-^124^I-FITC *in vivo*, BALB/c mice were intravenously injected with TAT-ΔNS3/4A-^124^I-FITC, and the radioactivity in serum samples collected at the indicated times was measured using a gamma-counter. *t_1/2_* = 2.55 min. Error bar: standard error of triplicate determinations.

To evaluate whether TAT-ΔNS3/4A-^124^I-FITC was selectivity retained in the NS3/4A-expressing cells, the HCC36 cells or NS3/4A-HCC36 cells were incubated with 37 kBq of TAT-ΔNS3/4A-^124^I-FITC probe at 37°C for 1 h in the presence or absence of the NS3/4A inhibitor telaprevir. After culturing for 0, 4, 6, and 8 h, the radioactivity retained in the cells was measured using a gamma-counter. A high level of radioactivity was detected in NS3/4A-HCC36 cells but not in control HCC36 cells, indicating TAT-ΔNS3/4A-^124^I-FITC was selectivity retained in the NS3/4A-expressing cells (Figure 4A). After inhibitor treatment, radioactivity did not accumulate in the NS3/4A-HCC36 cells, indicating that accumulation of the TAT-ΔNS3/4A-^124^I-FITC probe is dependent on the protease activity.

To evaluate the pharmacokinetics of TAT-ΔNS3/4A-^124^I-FITC in circulation, BALB/c mice were intravenously injected with TAT-ΔNS3/4A-^124^I-FITC and the radioactivity in serum samples collected at the indicated times was measured using a gamma-counter. The TAT-ΔNS3/4A-^124^I-FITC was rapidly eliminated from the blood following one-phase exponential decay kinetics with a half-life of 2.55 min (Figure 4B). Radioactivity in the blood was as low as 2.6 ± 0.1% ID/g at 4 h after injection, indicating that TAT-ΔNS3/4A-^124^I-FITC was rapidly cleared from circulation.

### Micro-PET imaging and biodistribution of TAT-ΔNS3/4A-^124^I-FITC *in vivo*

**FIGURE 5:**
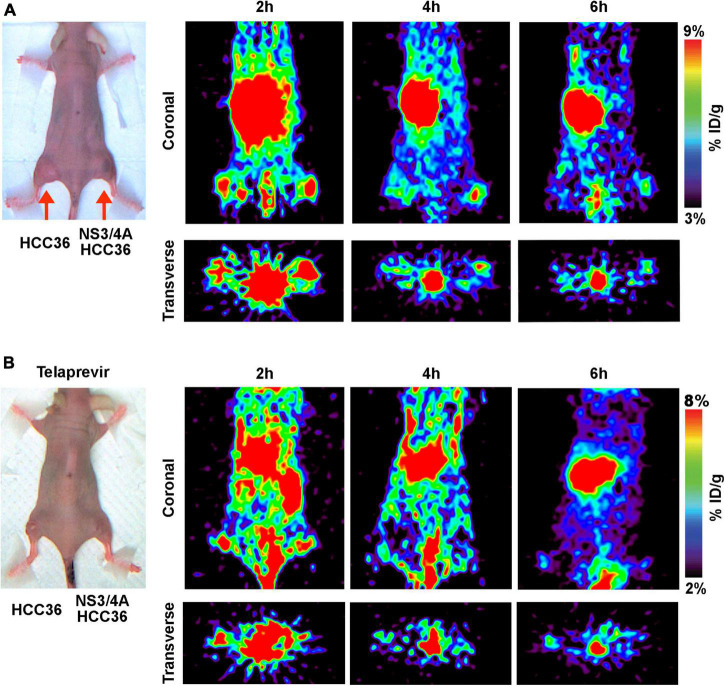
Micro-PET imaging of NS3/4A activity *in vivo*. **(A)** Mice bearing established NS3/4A-HCC36 (right hind leg) and HCC36 (left hind leg) tumors were injected with 3,700 kBq of TAT-ΔNS3/4A-^124^I-FITC. Coronal and transverse images were acquired at 2, 4, and 6 h after injection of the probe. **(B)** Mice bearing established NS3/4A-HCC36 (right hind leg) and HCC36 (left hind leg) tumors were intraperitoneally injected with telaprevir (20 mg/kg/day for 3 days) before intravenous injection of TAT-ΔNS3/4A-^124^I-FITC (3,700 kBq). Coronal images of tumor sections were acquired at 2, 4, and 6 h after injection of the probe.

To evaluate whether TAT-ΔNS3/4A-^124^I-FITC can be used to visualize NS3/4A activity *in vivo*, the NS3/4A-HCC36 or HCC36 tumor-bearing mice were intravenously injected with TAT-ΔNS3/4A-^124^I-FITC and imaged at 2, 4, and 6 h. Stronger radio-signals were detected in the NS3/4A-HCC36 tumors than in control HCC36 tumors (Figure 5A). Serial imaging analysis indicated that the highest image intensity occurred at 4 h after injection of TAT-ΔNS3/4A-^124^I-FITC. The radioactivity in the region of interest (ROI) was 1. 67-, 4. 42-, and 2.53-fold higher in NS3/4A-HCC36 tumors than in HCC36 tumors at 2, 4, and 6 h, respectively, suggesting that the TAT-ΔNS3/4A-^124^I-FITC was accumulated and retained in NS3/4A-HCC36 tumors. After inhibitor treatment, radioactivity did not accumulate in the NS3/4A-HCC36 tumors and the radioactivity was at a similar level in the NS3/4A-HCC36 and HCC36 tumors (Figure 5B). ROI in NS3/4A-HCC36 tumors was only 1. 13-, 0. 96-, and 1.31-fold greater than in HCC36 tumors at 2, 4, and 6 h, respectively. These results indicate that TAT-ΔNS3/4A-^124^I-FITC can be used to accurately represent NS3/4A protease activity *in vivo* using micro-PET.

**FIGURE 6:**
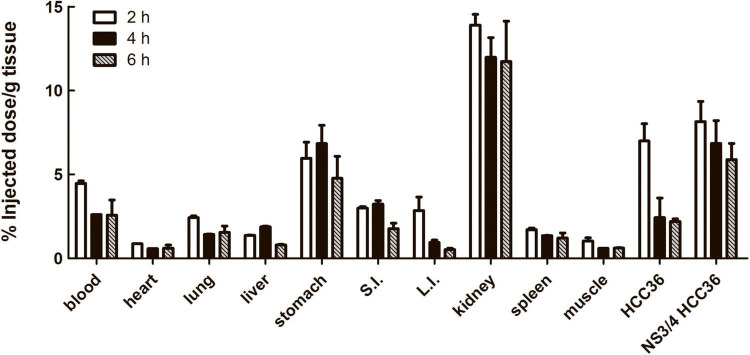
Biodistribution of TAT-ΔNS3/4A-^124^I-FITC in xenograft mice. Mice bearing established NS3/4A-HCC36 and HCC36 tumors were injected with 3,700 kBq of TAT-ΔNS3/4A-^124^I-FITC. Selected organs and tumors were removed from the mice after 2 (white column), 4 (black column), and 6 (gray column) h. The radioactivity of individual organs was measured using a gamma-counter and normalized for sample weights. The biodistribution of TAT-ΔNS3/4A-^124^I-FITC in selected organs was expressed as percentage injected dose/g tissue. Data represent mean ± SEM.

To investigate the biodistribution of TAT-ΔNS3/4A-^124^I-FITC *in vivo*, the NS3/4A-HCC36 or HCC36 tumor-bearing mice were intravenously injected with TAT-ΔNS3/4A-^124^I-FITC and then examined by measuring the radioactivity of the probe in organs at different time points after probe injection. Higher levels of radioactivity were detected in NS3/4A-HCC36 tumors than in control HCC36 tumors 2, 4, and 6 h after probe injection (Figure 6). The accumulation of radioactivity in NS3/4A-HCC36 tumors was 1. 6-, 3. 4-, and 2.7-fold higher than in HCC36 tumors at 2, 4, and 6 h, respectively. This result was similar to the micro-PET analysis. Notably, we also noted high levels of radioactivity were observed in the stomach and kidneys. However, the long-term bio-destruction results (Supplementary Figure 4) showed that the accumulation of probe in kidney would quickly reduce at 12 and 24 h, implying that the elimination of the radiolabeled probes was through the urinary system.

### Micro-PET imaging of NS3/4A activity in an orthotopic liver implantation tumor model

**FIGURE 7:**
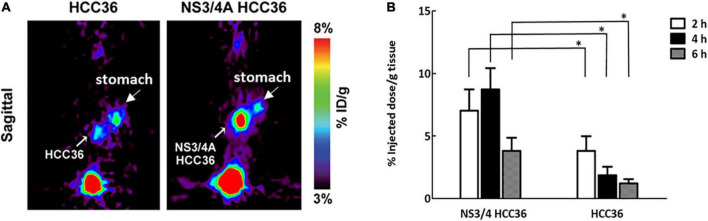
Micro-PET Imaging of NS3/4A activity in orthotopic liver implantation model. The NS3/4A-HCC36 tumors or HCC36 tumors from the ectopic tumors were harvested and transplanted into the left liver lobe of SCID mice (*n* = 3). After 2 weeks, the 3,700 kBq of TAT-ΔNS3/4A-^124^I-FITC was intravenously injected. **(A)** PET imaging was performed at 4 h after injection of the probe. **(B)** The accumulation of radioactivity in NS3/4A-HCC36 tumors and HCC36 tumors was measured using a gamma-counter at 2 (white column), 4 (black column), and 6 (gray column) h after probe injection. Data represent mean ± SEM. Student’s *t*-test analysis of data. Statistical analysis was compared NS3/4A-HCC36 with HCC36. *P* < 0.05 was considered statistically significant.

To further assess whether the TAT-ΔNS3/4A-^124^I-FITC can detect NS3/4A activity in deep liver tissue, we generated an orthotopic implantation tumor model in mice *via* surgery (Kim et al., 2009). The mice transplanted with NS3/4A-HCC36 tumors or control HCC36 tumors were intravenously injected with 3,700 kBq of TAT-ΔNS3/4A-^124^I-FITC and imaged *via* micro-PET. Radioactivity accumulated in NS3/4A-HCC36 tumors but not in control tumors 4 h after probe injection (Figure 7A). The ROI in NS3/4A-HCC36 tumors was 4.75-fold higher than in HCC36 tumors. In addition, the radioactivity of implanted NS3/4A-HCC36 tumors and HCC36 tumors was measured using a gamma-counter at 2, 4, and 6 h after probe injection. More radioactivity was detected in the implanted NS3/4A-HCC36 tumors than in control HCC36 tumors (Figure 7B). The uptake of radioactivity in implanted NS3/4A-HCC36 tumors was 1. 8-, 4. 7-, and 3.1-fold higher than in implanted HCC36 tumors at 2, 4, and 6 h, respectively, after probe injection. This result was similar to the micro-PET analysis. These results indicate that TAT-ΔNS3/4A-^124^I-FITC visualized by micro-PET imaging can be used to represent the NS3/4A activity in deep liver tissue.

## Discussion

We successfully developed a protease-activated retention peptide that allows real-time imaging of HCV NS3/4A protease activity by micro-PET. The *in vitro* and *in vivo* results demonstrated that TAT-ΔNS3/4A-FITC specifically accumulated in the NS3/4A protease-expressing area. Furthermore, PET imaging results showed that the NS3/4A protease activity can be systemically detected in xenograft mice or in orthotopic liver-implanted mice. These results indicate that TAT-ΔNS3/4A-^124^I-FITC may provide a probe that allows visualization of NS3/4 activity by PET technology to systemically track protease activity and distribution *in vivo*.

The technology to tracking the NS3 protease activity *in vivo* would provide a powerful tool to design the personalized protease inhibitor-based therapies and to monitor the development of liver cirrhosis and hepatocellular carcinoma. Currently, HCV is commonly diagnosed by reverse transcription-PCR (RT-PCR) or quantitative PCR to detect the HCV RNA in patient blood. However, the viral distribution and protease activity cannot be detected by RT-PCR or anti-HCV ELISA in patients. Liver-biopsy is, therefore, currently the best approach to detect the viral distribution (Carreno et al., 2004; Castillo et al., 2004) or viral protease activity in liver (Chao et al., 2011). However, liver biopsy is the partial and invasive method that only detects the infection in liver and may increase the risk of liver damage. The HCV also can infect the extra-hepatic tissue and can increase the incidence of several diseases, including autoimmune disorders (Yang et al., 2014), diabetes (Arase et al., 2009), nervous disorders (Mariotto et al., 2014), and chronic kidney disease (Sumida et al., 2010), indicating the systemically image system is the suitable approach to detect the distribution of NS3/4A protease activity. Here, our results show that our probe could be used to specifically detect the protease activity in NS3/4A protease-expressing cells/tumors and in liver-implanted tumors *via* a PET imaging system. These results indicated that TAT-ΔNS3/4A-^124^I-FITC may provide a clinically available PET probe to represent NS3/4A activity to systemically track protease activity and distribution *in vivo*.

Development of a clinically available imaging probe to detect cellular protease activity has potential applications in many diseases. Overexpression of cellular proteases has been reported to play an important role in many diseases, including various viral infections (Carreno et al., 2004; Castillo et al., 2004; Lee et al., 2004), Alzheimer’s disease (Lescar et al., 2008), and cancers (Brik and Wong, 2003; Vermehren and Sarrazin, 2011). Various imaging strategies aiming to evaluate protease activity have been developed. For example, a quenched near-infrared fluorescence (NIRF) probe was developed to track human immunodeficiency virus specific protease (HIV PR) activity *in vivo* (Ta et al., 2013). Various protease-activation bioluminogenic probes have been designed using a similar strategy to image the furin activity in breast tumors (Dragulescu-Andrasi et al., 2009) or to detect caspase 3 activity in gliomas (Shah et al., 2005). However, the shallow penetration of the bioluminogenic imaging still limits its use in the clinic (Cheng et al., 2012; Chuang et al., 2012). Here, we successfully developed a clinically available PET probe to image NS3/4A activity. The protease substrate of this probe can be changed to allow imaging of other cellular proteases relevant to different diseases. Furthermore, the hydrophilic domain can be easily conjugated to a variety of contrast agents, such as^124^ I for PET (Pentlow et al., 1996), ^111^In-DOTA for SPECT, or Gd-DOTA for MRI, giving flexibility in choice of imaging system.

Development of a quickly metabolic and low toxicity probe is very important. The quickly metabolic probe usually be considered has the low toxicity and low side effects. Theoretically, peptide-based probe is metabolized briefly into ammonium and carbon backbone, eventually enter urea cycle and TCA cycle. For example, Chuang and colleagues developed a PEG-peptide-^18^F-TMR probe to image the *in vivo* MMP protease activity *in vivo* by PET (Park et al., 2012). They also reported this based probe was quickly the elimination of the radiolabeled probes in urine. Our long-term bio-destruction results (Supplementary Figure 4) also indicated that the non-specific accumulation of probe in kidney and in stomach at 4 h, and the accumulation of probe in kidney and in stomach would quickly reduce at 12 and 24 h. The result also indicated the radioactivity of probe did not accumulate in the all of tissues at 24 h. These results implied that this probe will be rapidly metabolized and eliminated out of the body through the urinary system. In addition, many studies have been reported that the deiodination of probe would usually happen *in vivo* and that would cause the non-specific accumulation in stomach (Koehler et al., 2010; Oh and Ahn, 2012). We considered that was the reason why our probe would non-specifically accumulate in stomach. However, the result also indicated the radioactivity of probe did not accumulate in the all of tissues at 24 h, implying that this probe will be rapidly metabolized and eliminated out of the body through the urinary system. We also have detected the *in vitro* toxicity of our probe, and we treated the human embryonic kidney cell (HEK293) with different concentrations (0.1–2,000 ug/ml) of probe. The results (Supplementary Figure 5) indicated the different concentrations (0.1–2,000 ug/ml) of probe could not inhibit the growth of HEK293 as compared with CPT-11(anti-cancer drug), indicating this probe has the very low toxicity. Therefore, we considered that our probe is quickly metabolic probe, and low toxicity might be more suitable for clinical imaging.

The strategy may be extended to image other cellular proteases in different diseases *via* multimodality system in clinic. Therefore, we hypothesized that the mechanism of cellular clearance of the probe was through (1) directly interaction with inner member and (2) the exocytosis of the TAT peptide contained endosomes. For example, Dr. Rayne has reported the cellular Tat could bind to the phosphatidylinositol-(4,5)-bisphosphate in the inner member, resulting in Tat insertion into the plasma membrane, and then enables efficiently secretion of HIV1 from cytoplasm into medium (Rayne et al., 2010), suggesting the cellular Tat would interact with membrane and exclude from the cells. In addition, several studies have shown the uptake mechanism of arginine rich CPPs, such as TAT peptide or R9 peptide, was dependent on the endocytotic pathways (Rubin et al., 1992; Richard et al., 2003, 2005). After internalization, the TAT peptide would have trapped in endosomes of cells (Ross and Murphy, 2004; Debaisieux et al., 2012) and might slowly diffuse into cytoplasm. Therefore, we considered that the cellular TAT peptide in the endosomes might be excluded from cell *via* exocytosis, such as the mechanism of TAT-derived HIV to cross the BBB and to reach the brain (Banks et al., 2005; Mahajan et al., 2008; Cooper et al., 2012). Therefore, we hypothesized that the mechanism of cellular clearance of the probe was through (1) directly interaction with inner member and (2) the exocytosis of the TAT peptide contained endosomes.

This NS3/4A-activatable PET probe could real-time imaging of NS3/4A protease activity *in vivo*. This strategy would provide several advantages: (1) The probe has high penetrability for imaging of NS3/4A activity in deep tissues; (2) the possibility of changing the substrate potentially allows the generation of a wide range of probes for other cellular proteases; (3) this is a multimodality system that can be easily conjugated with a variety of contrast agents; (4) this probe provides a convenient platform for the development of protease inhibitors in human or big animal studies. Based on these advantages, the TAT-ΔNS3/4A-^124^I-FITC probe potentially provides a clinical available approach to determine the protease distribution and optimize protease inhibitor-based therapy.

## Data availability statement

The original contributions presented in this study are included in the article/Supplementary material, further inquiries can be directed to the corresponding authors.

## Ethics statement

This animal study was reviewed and approved by Affidavit of Approval of Animal Use Protocol Kaohsiung Medical University, Center for laboratory animals, Kaohsiung Medical University.

## Author contributions

M-YH and J-CL: conception and design. C-HC, T-LC, Yi-JH, J-CL, H-EW, W-CC, C-CK, and M-YH: development of methodology. C-HC, J-CL, W-CC, H-EW, T-LC, Y-CL, Yi-JH, Y-CH, W-WL, Ya-JH, C-CK, K-CH, and M-YH: acquisition of data. C-HC, J-CL, Yi-JH, and M-YH: analysis and interpretation of data. C-HC, M-YH, Yi-JH, H-EW, J-CL, and T-LC: writing, review, and/or revision of the manuscript. H-EW, T-LC, and J-CL: administrative, technical, or material support. C-HC, J-CL, and M-YH: study supervision. All authors contributed to the article and approved the submitted version.

## Supplementary material

Supplementary Figure 1 Characterization of the expression and function of NS3/4A-expressing cells. We constructed the NS3/4A gene into a lentiviral vector, pLKO_AS3 NS3/4A, to directly express the HCV NS3/4A protease in human hepatocellular carcinoma HCC36 cells. **(A)** HCC36, NS3/4A-HCC36, and HCV replicon-containing cells (AVA5) were harvested to detect the expression HCV NS3. Western blotting was performed using anti-HCV NS3 (ab13830, 1:1000, Abcam) and anti-GAPDH antibodies (GTX100118, 1:1000, GeneTex). GAPDH was used as a loading control. **(B)** HCC36, NS3/4A-HCC36, and AVA5 were transfected with NS3 response reporter vector Peg (DEΔ4AB) SEAP, containing the NS4A/B junction between *egfp* and *seap*. After 3 days, total cell supernatant was analyzed for SEAP activity according to the manufacturer’s instructions. Each value represents the mean fold ± SD of triplicate experiments after normalization of luciferase activities. The error bars denote SD of the mean.

Supplementary Figure 2 Characterization of the function of NS3/4A in virus-infected cells. HCC36, NS3/4A-HCC36, and JHF-1-infected cells were transfected with NS3 response reporter vector pEG (DEΔ4AB) SEAP, containing the NS4A/B junction between egfp and seap. After 3 days, total cell supernatant was analyzed for SEAP activity according to the manufacturer’s instructions. Each value represents the mean fold ± SD of triplicate experiments after normalization of luciferase activities. The error bars denote SD of the mean.

Supplementary Figure 3 HPLC chromatograms of **(A)** Na^124^I solution, **(B)** crude product of TAT-ΔNS3/4A-^124^I-FITC, and **(C)** final product TAT-ΔNS3/4A-^124^I-FITC: radio-peak of ^124^I and TAT-ΔNS3/4A-^124^I-FITC. The retention time of ^124^I and TAT-ΔNS3/4A-^124^I-FITC was ∼4.7–5 and 6.7 min, respectively.

Supplementary Figure 4 Long-term biodistribution of TAT-ΔNS3/4A-^124^I-FITC in xenograft mice. Mice were injected with 3,700 kBq of TAT-ΔNS3/4A-^124^I-FITC. Selected organs and tumors were removed from the mice after 4, 12, and 24 h. The radioactivity of individual organs was measured using a gamma-counter and normalized for sample weights. The biodistribution of TAT-ΔNS3/4A-^124^I-FITC in selected organs was expressed as percentage injected dose/g tissue. Data represent mean ± SEM.

Supplementary Figure 5 To evaluate the specific retention of probe *via* flow cytometry, we used the WT probe (GRKKRRQRRR-DEDEDEDEMEECASH-LKKKYK-FITC) and the scrambled sequence probe (GRKKRRQRRR-ECEEEEESMDD DAH-LKKKYK) that could not be activated by protease as a control group to test the specific retention of probe *via* flow cytometry. HCC36 cells, NS3/4A-HCC36 cells, or JFH-1-infected HCC36 cells were incubated with 10 μM TAT-ΔNS3/4A-FITC or 10 μM TAT-scrambled NS3/4A-FITC in the absence or presence of 2 μM telaprevir (NS3/4A protease inhibitor) at 37°C for 1 h. The cells were washed with DMEM containing 10% serum three times per hour. After culturing for 1 or 8 h, the fluorescence of viable cells was observed *via* flow cytometry.

Supplementary Figure 6 *In vitro* cytotoxicity of TAT-ΔNS3/4A-FITC. HEK293 cells were incubated with different concentrations (0.1–2,000 ug/ml) of TAT-ΔNS3/4A-FITC or CPT-11. After incubation for 72 h, cell viability was measured *via* the rate of cellular ATP synthesis to detect the cytotoxicity of TAT-ΔNS3/4A-FITC for HEK293 cells. CPT-11: irinotecan hydrochloride.
