# Supplementary figures and images for: Micro-PET imaging of hepatitis C virus NS3/4A protease activity using a protease-activatable retention probe
Source: Front Microbiol. 2022 Nov 4;13:896588. doi: 10.3389/fmicb.2022.896588 (PMC9672079; doi:10.3389/fmicb.2022.896588)

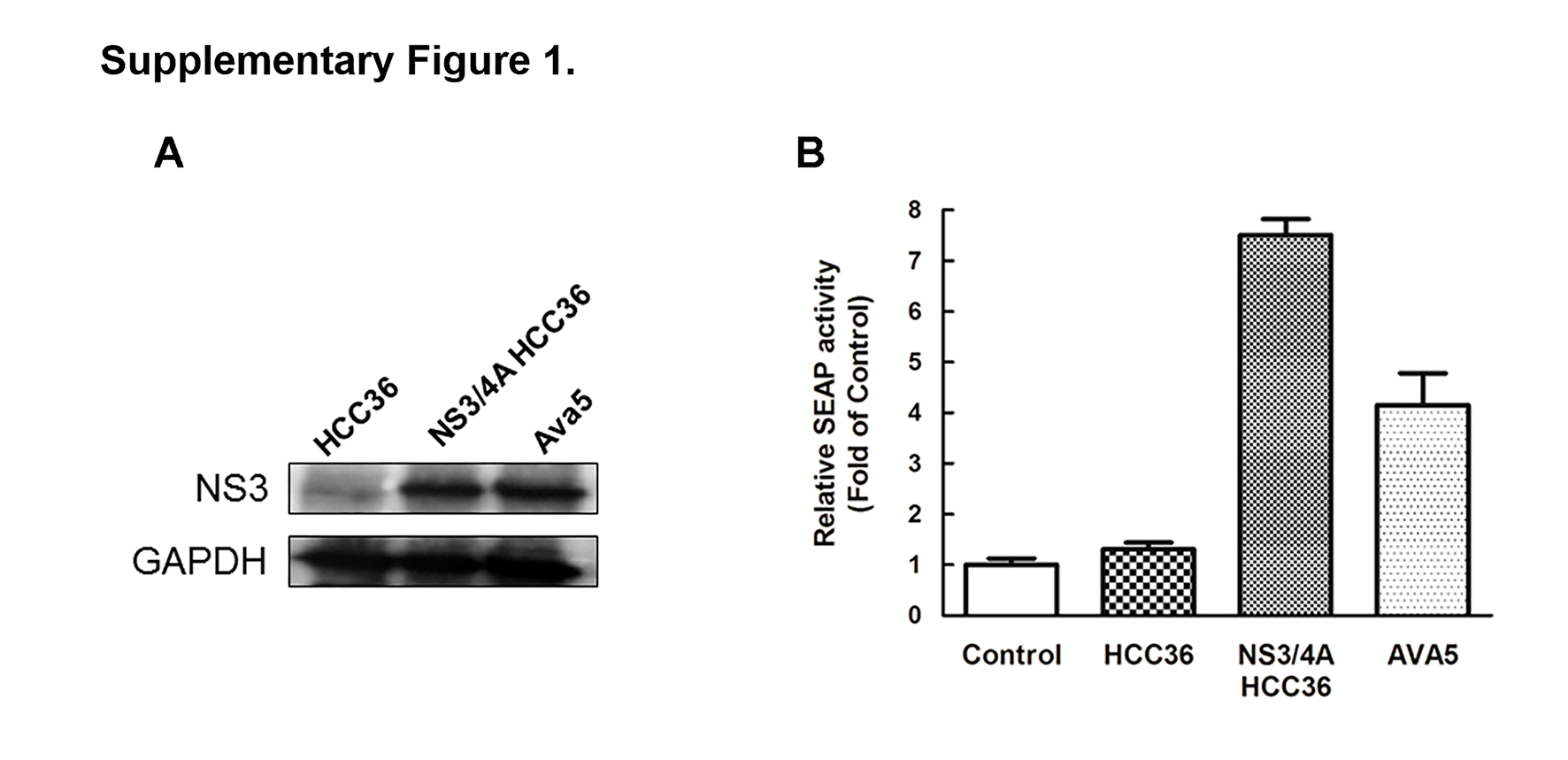

Supplement: Supplementary Figure 1 — Characterization of the expression and function of NS3/4A-expressing cells. We constructed the NS3/4A gene into a lentiviral vector, pLKO_AS3 NS3/4A, to directly express the HCV NS3/4A protease in human hepatocellular carcinoma HCC36 cells. (A) HCC36, NS3/4A-HCC36, and HCV replicon-containing cells (AVA5) were harvested to detect the expression HCV NS3. Western blotting was performed using anti-HCV NS3 (ab13830, 1:1000, Abcam) and anti-GAPDH antibodies (GTX100118, 1:1000, GeneTex). GAPDH was used as a loading control. (B) HCC36, NS3/4A-HCC36, and AVA5 were transfected with NS3 response reporter vector Peg (DEΔ4AB) SEAP, containing the NS4A/B junction between egfp and seap. After 3 days, total cell supernatant was analyzed for SEAP activity according to the manufacturer’s instructions. Each value represents the mean fold ± SD of triplicate experiments after normalization of luciferase activities. The error bars denote SD of the mean. [file Image_1.TIF]

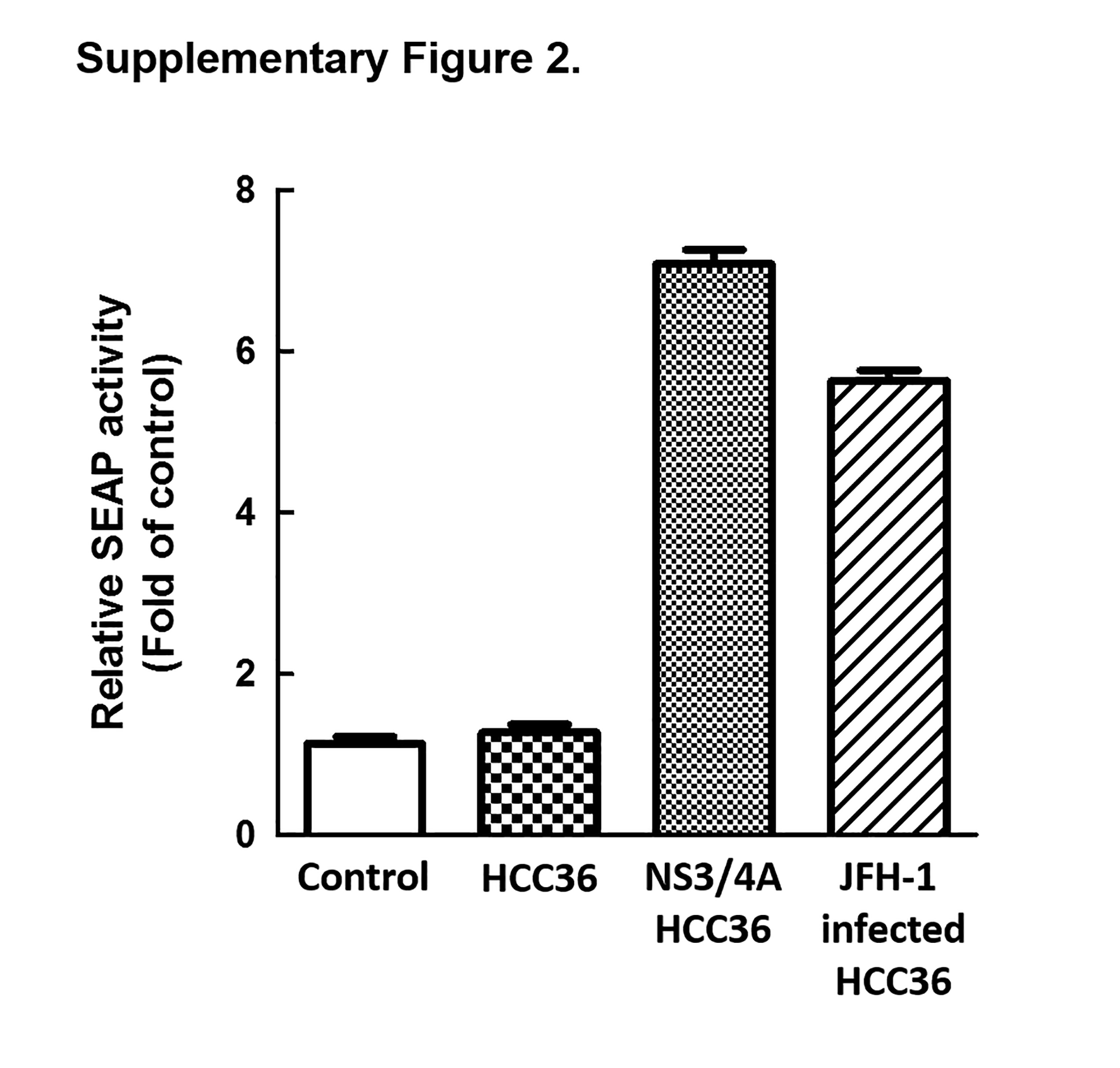

Supplement: Supplementary Figure 2 — Characterization of the function of NS3/4A in virus-infected cells. HCC36, NS3/4A-HCC36, and JHF-1-infected cells were transfected with NS3 response reporter vector pEG (DEΔ4AB) SEAP, containing the NS4A/B junction between egfp and seap. After 3 days, total cell supernatant was analyzed for SEAP activity according to the manufacturer’s instructions. Each value represents the mean fold ± SD of triplicate experiments after normalization of luciferase activities. The error bars denote SD of the mean. [file Image_2.TIF]

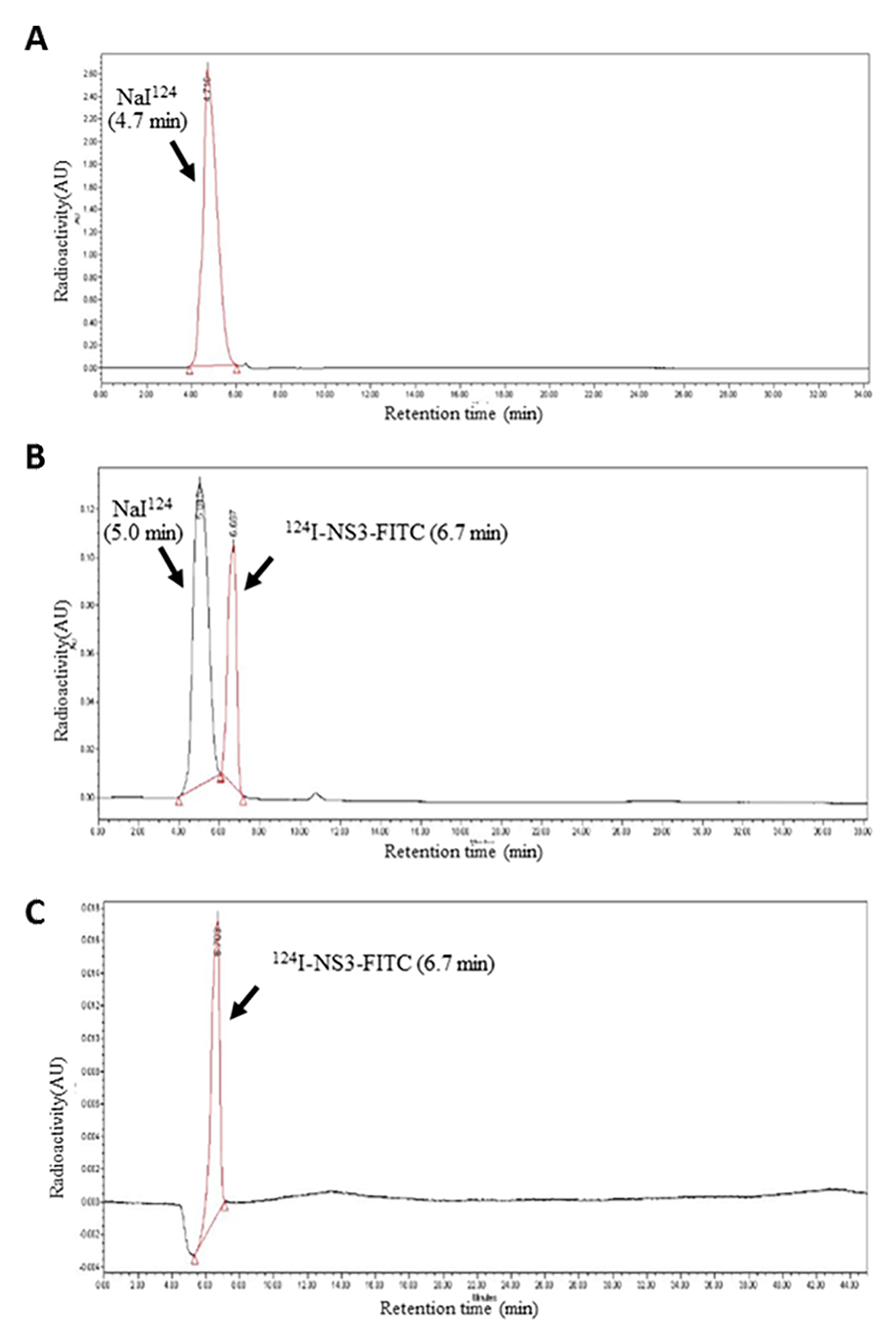

Supplement: Supplementary Figure 3 — HPLC chromatograms of (A) Na124I solution, (B) crude product of TAT-ΔNS3/4A-124I-FITC, and (C) final product TAT-ΔNS3/4A-124I-FITC: radio-peak of 124I and TAT-ΔNS3/4A-124I-FITC. The retention time of 124I and TAT-ΔNS3/4A-124I-FITC was ∼4.7–5 and 6.7 min, respectively. [file Image_3.TIF]

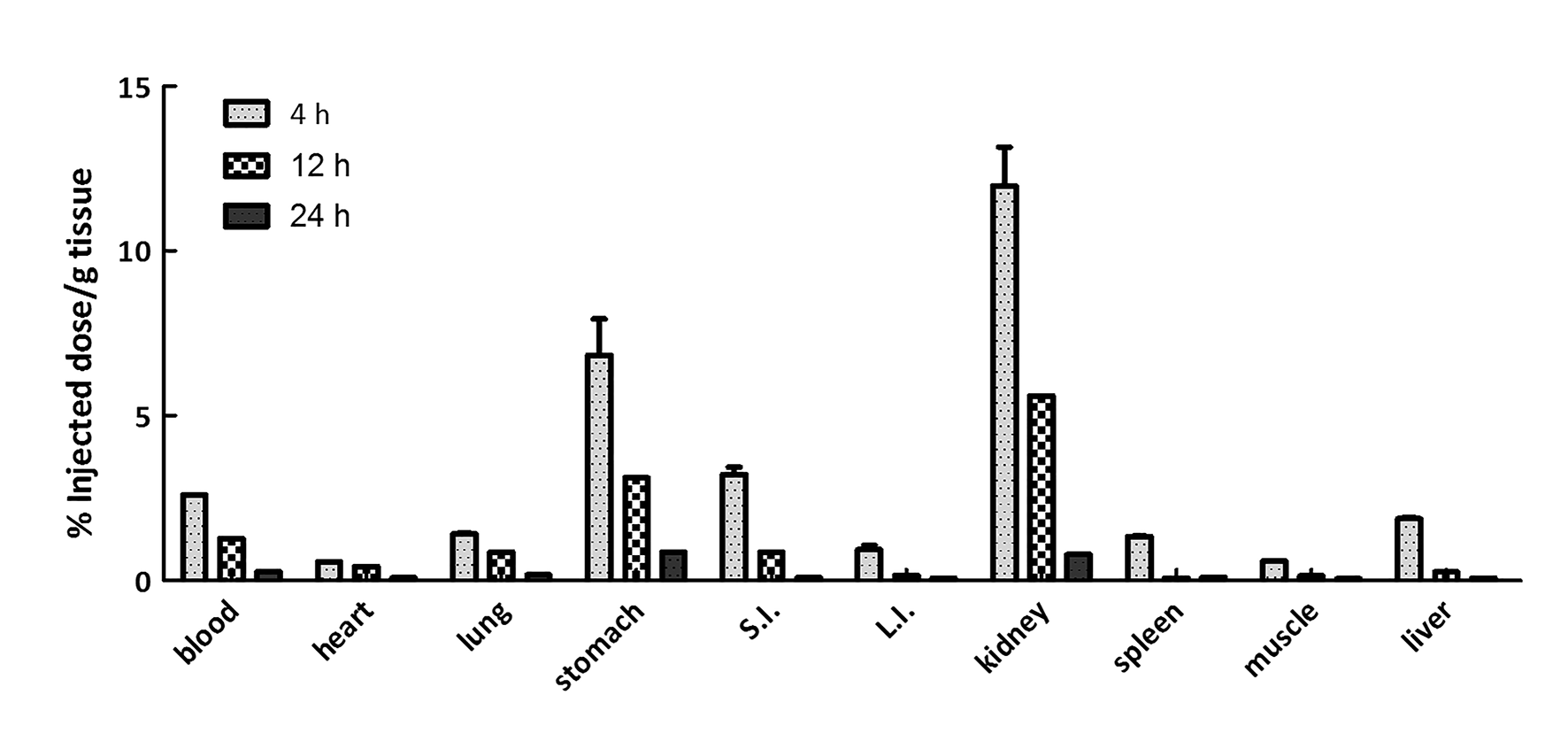

Supplement: Supplementary Figure 4 — Long-term biodistribution of TAT-ΔNS3/4A-124I-FITC in xenograft mice. Mice were injected with 3,700 kBq of TAT-ΔNS3/4A-124I-FITC. Selected organs and tumors were removed from the mice after 4, 12, and 24 h. The radioactivity of individual organs was measured using a gamma-counter and normalized for sample weights. The biodistribution of TAT-ΔNS3/4A-124I-FITC in selected organs was expressed as percentage injected dose/g tissue. Data represent mean ± SEM. [file Image_4.TIF]

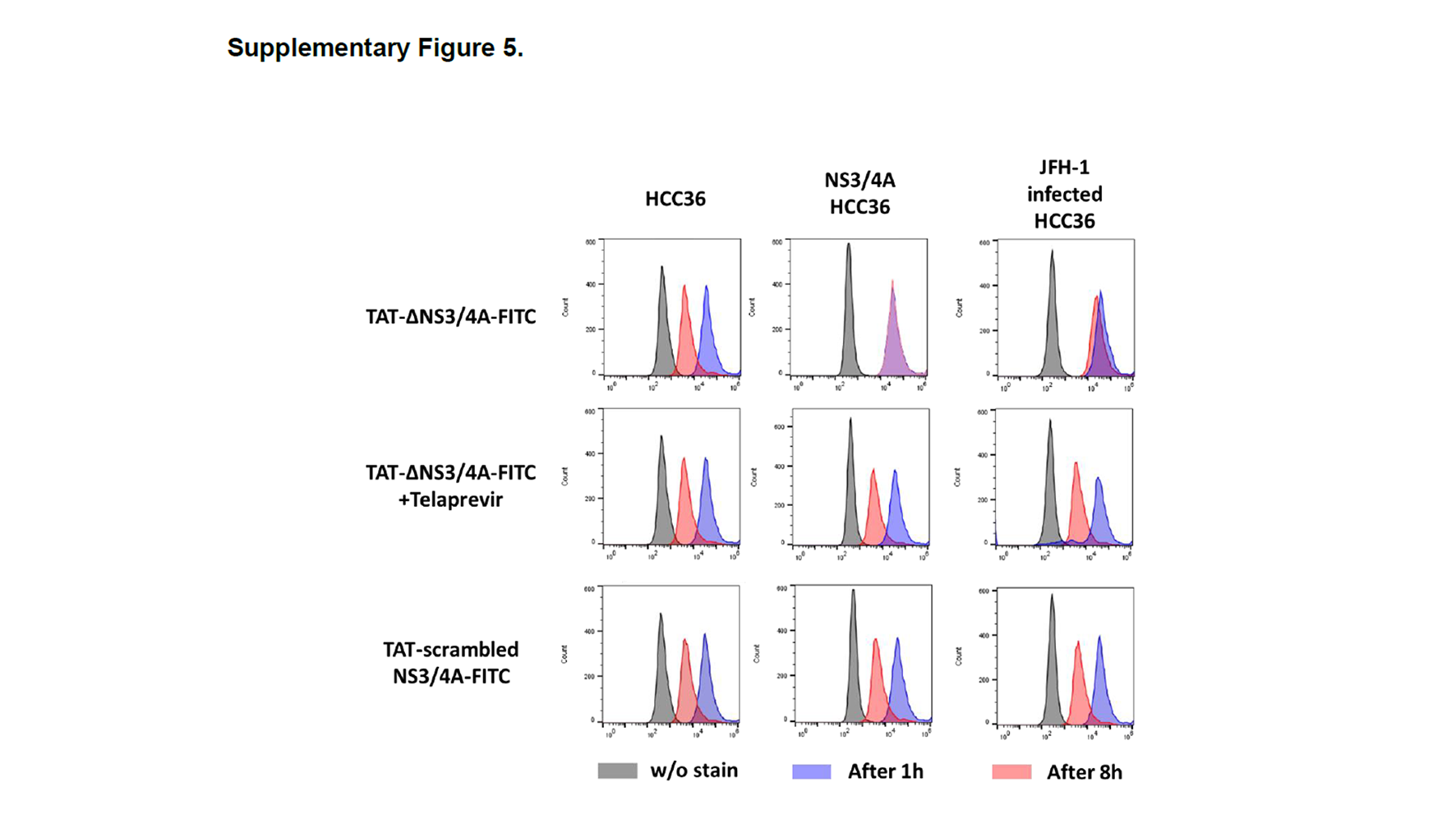

Supplement: Supplementary Figure 5 — To evaluate the specific retention of probe via flow cytometry, we used the WT probe (GRKKRRQRRR-DEDEDEDEMEECASH-LKKKYK-FITC) and the scrambled sequence probe (GRKKRRQRRR-ECEEEEESMDD DAH-LKKKYK) that could not be activated by protease as a control group to test the specific retention of probe via flow cytometry. HCC36 cells, NS3/4A-HCC36 cells, or JFH-1-infected HCC36 cells were incubated with 10 μM TAT-ΔNS3/4A-FITC or 10 μM TAT-scrambled NS3/4A-FITC in the absence or presence of 2 μM telaprevir (NS3/4A protease inhibitor) at 37°C for 1 h. The cells were washed with DMEM containing 10% serum three times per hour. After culturing for 1 or 8 h, the fluorescence of viable cells was observed via flow cytometry. [file Image_5.TIF]

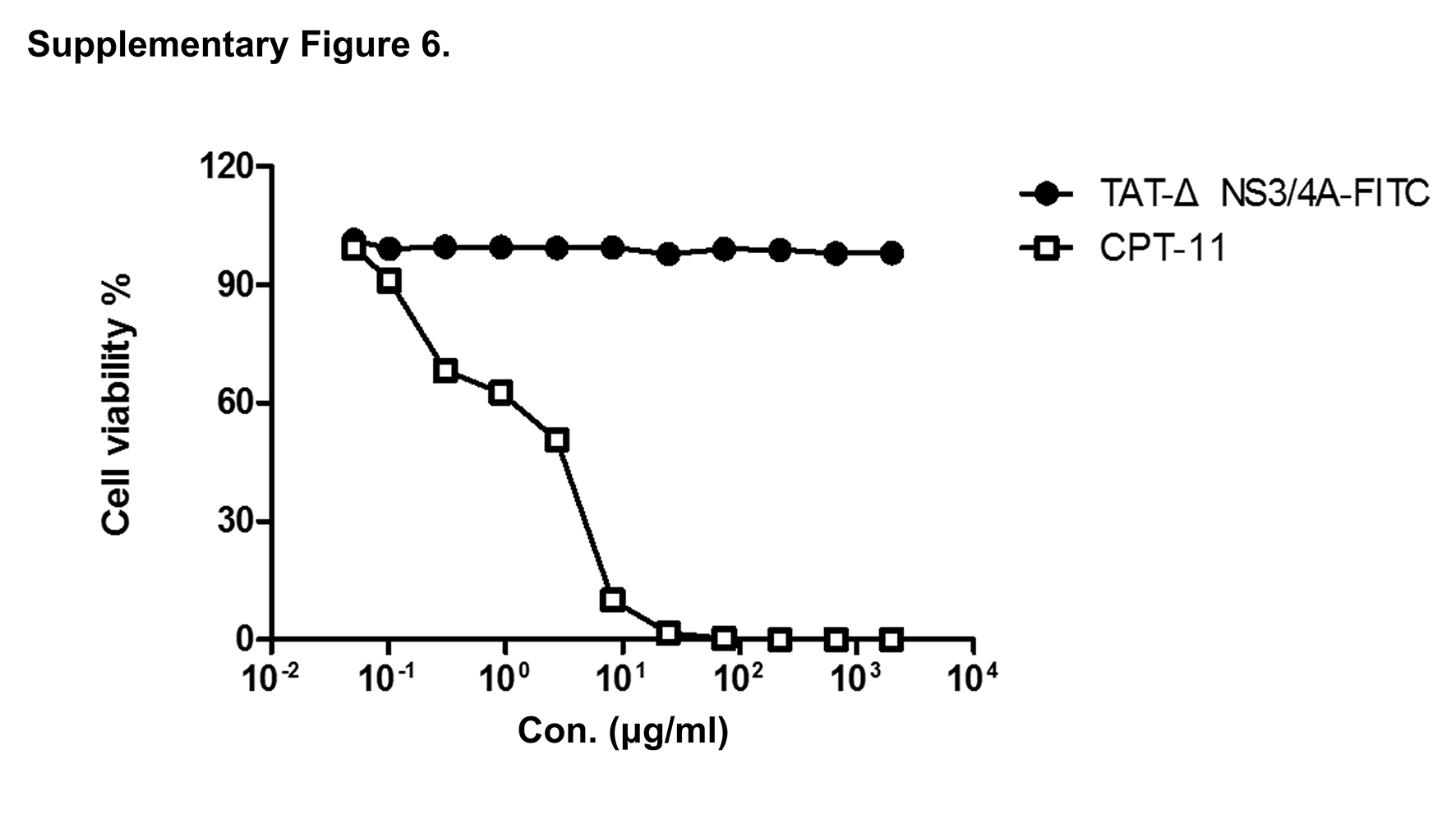

Supplement: Supplementary Figure 6 — In vitro cytotoxicity of TAT-ΔNS3/4A-FITC. HEK293 cells were incubated with different concentrations (0.1–2,000 ug/ml) of TAT-ΔNS3/4A-FITC or CPT-11. After incubation for 72 h, cell viability was measured via the rate of cellular ATP synthesis to detect the cytotoxicity of TAT-ΔNS3/4A-FITC for HEK293 cells. CPT-11: irinotecan hydrochloride. [file Image_6.TIF]
